# Exploratory behavior undergoes genotype–age interactions in a wild bird

**DOI:** 10.1002/ece3.5430

**Published:** 2019-07-31

**Authors:** Barbara Class, Jon E. Brommer, Kees van Oers

**Affiliations:** ^1^ Department of Biology University of Turku Turku Finland; ^2^ Department of Animal Ecology Netherlands Institute of Ecology (NIOO‐KNAW) Wageningen The Netherlands

**Keywords:** animal model, development, genotype–age interaction, heritability, personality, plasticity

## Abstract

Animal personality traits are often heritable and plastic at the same time. Indeed, behaviors that reflect an individual's personality can respond to environmental factors or change with age. To date, little is known regarding personality changes during a wild animals' lifetime and even less about stability in heritability of behavior across ages. In this study, we investigated age‐related changes in the mean and in the additive genetic variance of exploratory behavior, a commonly used measure of animal personality, in a wild population of great tits. Heritability of exploration is reduced in adults compared to juveniles, with a low genetic correlation across these age classes. A random regression animal model confirmed the occurrence of genotype–age interactions (G×A) in exploration, causing a decrease in additive genetic variance before individuals become 1 year old, and a decline in cross‐age genetic correlations between young and increasingly old individuals. Of the few studies investigating G×A in behaviors, this study provides rare evidence for this phenomenon in an extensively studied behavior. We indeed demonstrate that heritability and cross‐age genetic correlations in this behavior are not stable over an individual's lifetime, which can affect its potential response to selection. Because G×A is likely to be common in behaviors and have consequences for our understanding of the evolution of animal personality, more attention should be turned to this phenomenon in the future work.

## 1 INTRODUCTION

Behaviors are labile traits, which by definition can be adjusted by individuals in response to variations in external (biotic, abiotic) factors but also internal factors such as age. At the same time, behavioral responses often consistently differ between individuals across a wide variety of taxa (Bell, Hankison, & Laskowski, 2009). These consistent individual differences in behavior are assumed to capture aspects of animal personality (Réale, Reader, Sol, McDougall, & Dingemanse, 2007) and, importantly, are often caused by additive genetic variation (Dochtermann, Schwab, & Sih, 2015; van Oers & Mueller, 2010). Hence, heritable behaviors capture animal personality, can respond to selection, and studying additive genetic variation in behaviors is essential to better understand their evolutionary potential and the maintenance of their variation in populations, a central theme in animal personality research.

Behavioral ecologists are increasingly following the lead of human psychologists and animal breeders in studying animal personality from a lifetime perspective (Stamps & Groothuis, 2010a, 2010b). Age‐related changes in behaviors can be seen as behavioral plasticity “in response to” age and therefore can be seen on the population, individual, and genetic levels (Brommer, 2013). For example, a behavior shows age‐related plasticity when (assuming no selective disappearance) the population mean behavior changes with age. This occurs when individuals change their behavior during their lifetime. Importantly, individuals within a population can vary in the way their behavior changes with age (individual–age interaction, I×A) and these plastic changes can be heritable (genotype–age interaction, G×A). Both I×A and G×A act to break down the correlation of behavior between ages as they imply that individuals with high values at younger ages may not have high values at later ages. In fact, behavioral cross‐age correlations are typically low (0.3) (Brommer & Class, 2015) implying that I×A/G×A are common.

Importantly, G×A is a process that can impact the evolution of any trait by inducing changes in heritability across ages and genetic correlations between traits, thereby altering their responses to selection (Lynch & Walsh, 1998). This process is also particularly interesting in the context of aging because it is considered as the “fingerprint” of evolved senescence (Charmantier, Brommer, & Nussey, 2014) and age‐related changes in additive genetic variance of life history traits have been reported in wild animal populations (reviewed in Charmantier et al., 2014) and in plants (Pujol, Marrot, & Pannell, 2014). In contrast, despite phenotypic and individual patterns suggesting G×A in behaviors to be common, this phenomenon remains understudied in the context of animal personality research. To date, studies investigating age‐related changes in heritability or explicitly testing for G×A in behavior remain scarce (Class & Brommer, 2015, 2016). Indeed, detecting G×A in any trait requires measures of relatives at various ages in populations and sufficiently large datasets, which, for wild populations, implies substantial collection efforts and long‐term monitoring. This type of data may not be abundant in animal personality research, where performing behavioral tests can be particularly demanding and priority is given to repeated measures of individuals (Niemelä & Dingemanse, 2018) rather than measures of relatives. This lack of studies on G×A in animal personality is unfortunate, not only because knowledge of an evolutionarily relevant process in an important part of individual phenotypes is lacking, but also because age‐related changes in heritability and genetic correlations can induce changes in repeatability and behavioral syndromes (van Oers & Sinn, 2011) and thus challenge what defines animal personality.

In the present paper, we investigate age‐related plasticity and genetic variation in age‐related plasticity in exploratory behavior, a commonly used measure of animal personality in a wild population of great tits (*Parus major*), using a quantitative genetic framework. Earlier studies found exploratory behavior to be heritable in this population (Drent, Oers, & Noordwijk, 2002 for juveniles, Dingemanse, Both, Drent, Oers, & Noordwijk, 2002 all individuals up to 1 year old, Santure et al., 2015 all ages) and in other great tit populations ( Korsten, Overveld, Adriaensen, & Matthysen, 2013; Nicolaus, Piault, Ubels, Tinbergen, & Dingemanse, 2016; Quinn, Patrick, Bouwhuis, Wilkin, & Sheldon, 2009; all ages). However, no study has estimated heritability of exploration behavior in juveniles and adults separately or the genetic correlation in exploration across ages, both of which are aspects of G×A. First, we use a character‐state approach to test whether quantitative genetic parameters of exploration behavior differ between juveniles (<1 year old) and adult birds and to calculate the genetic correlation across these two life stages. We find that exploration is heritable in juvenile birds but not in adults, and the presence of G×A is confirmed by a reaction norm approach. In particular, we show that additive genetic variance decreases to become nonsignificantly different from zero before individuals reach their first year and that this rapid reduction in additive genetic variance is not due to the selective disappearance of certain juveniles based on their exploration.

## 2 MATERIAL AND METHODS

### 2.1 Population studied

Data were collected in years 1998–2017 in the nest box populations Westerheide and Boslust in the “Groot Warnsborn” area, nearby Arnhem in the Netherlands (5°84′E, 52°01′N). This area consists of plots of mixed pine and deciduous forest with small open patches, in total covering approximately 200 ha and containing about 400 nest boxes distributed in a regular grid. The population was monitored throughout the year. Starting early April, nest boxes were checked weekly for nesting activity. When nesting material was observed, the frequency was increased to twice a week. First egg laying date, clutch size and hatching date were recorded. When nestlings were 10 days old, the parents were caught using spring traps in the nest box entrance. Parents were ringed when they did not have an individual ring yet, and their age was determined based on the coloration of their greater coverts (as yearling or older). For locally fledged birds, age was calculated (in months) based on their hatching date. Birds for which hatching date was unknown were assumed to have hatched in May. Nestlings were weighed and ringed at day 14 after hatching. Several days after fledging, nest boxes were checked for fledging and unfledged chicks were noted.

### 2.2 Behavioral assay

Birds were captured at maintained feeding sites between June and April using mist nets, or during evening roost inspections between November and March. Birds that were not tested before were transported to the bird housing facilities of the Netherlands Institute of Ecology (NIOO‐KNAW). They were kept in single cages of 90 × 50 × 50 cm until behavioral testing the next morning and provided with live mealworms, sunflower seeds, pieces of apple, and water. The behavioral assay measuring exploratory behavior consisted of an open‐field test in a novel testing room (4.0 × 2.5 × 2.5 m) that contained five artificial trees. Before the test, the holding cage was darkened and a sliding door that provided access to the test room was opened. By switching on the light in the testing room, the bird was let in the room without handling. The bird was observed from behind a one‐way screen, and the total number of movements in the first two minutes after a bird entered was measured (for more details on the behavioral assay see Dingemanse et al., 2002). After behavioral tests, birds were released around their area of catching.

### 2.3 Population pedigree

Exploration scores and age were available for 5,144 individuals, which were connected through a pedigree. The pruned pedigree (where uninformative individuals are excluded) holds record for 6,618 individuals, 2,434 maternities, 2,627 paternities, 2,863 full sibs, 3,823 maternal sibs, 4,334 paternal sibs, 960 maternal half‐sibs, 1,471 paternal half‐sibs, a mean family size of 2.2, a mean pairwise relatedness of 3.7e‐04, and a maximum pedigree depth of 11.

### 2.4 Univariate and bivariate animal models

To estimate heritability in exploratory behavior as well as genetic correlations across sexes and age classes, we fitted univariate and bivariate animal models, which are mixed models using the relatedness between individuals as derived from the pedigree (Wilson et al., 2009). The structure of the model is the following:

$$y=X\beta +Z_{A}u_{A}+\sum Z_{k}u_{k}+e$$

where ***y*** is a vector of all the information on all the individuals, *β* is a vector of one or more fixed effects, and ***X*** is a design matrix relating the appropriate fixed effects to each individual. In contrast to earlier studies on exploratory behavior in this population, we used raw exploration scores as the response variable instead of corrected exploratory scores (corrected for seasonal effects). This is because the animal model allows correcting directly for seasonal effects by fitting them in the fixed effect part of the model, contrary to other statistical approaches used earlier (see Dingemanse et al., 2002). In all models, sex and the quadratic effects of age (in months) and of date of measurement (in June days) were fitted as fixed effects. In the random effect part of the model, $u_{A}$ is the vector of additive genetic effects and $Z_{A}$ is the design matrix relating the appropriate additive genetic effect to each individual. The summation $\sum Z_{k}u_{k}$ allows for more random components such as year of measurement and the observer identity. Finally, ***e*** is a vector of residual errors. Additive genetic effects and residuals were assumed to be (bivariate) normally distributed with a mean of zero and variances to be estimated. The matrix **G** (for vector$u_{A}$) and its elements (one variance in the univariate model, two variances, and a covariance in the bivariate model) was estimated using information on the coefficient of coancestry Θ*_ij_* between individuals *i* and *j*, derived from the pedigree. Animal models were solved using Restricted Maximum Likelihood (REML), as implemented in ASReml‐R (Butler, Cullis, Gilmour, & Gogel, 2009; VSN International). Heritability (*h* ^2^) was expressed by the ratio *V*_A_/*V*_P_ where *V*_P_, the phenotypic variance, is defined as the sum of the REML estimates of additive genetic effects and residuals (*V*_A_ and *V*_R_, respectively) and is hence conditional on the fixed effect structure of the model. Statistical significance of fixed effects was based on conditional Wald *F* tests.

Most individuals in this dataset were measured only once, and we used the first exploratory behavior score for the few individuals with repeated‐measures. We used a bivariate animal model to show that the genetic correlation between sexes is not different from 1 and sex‐specific additive genetic variances do not differ, using a Likelihood Ratio Test (LRT) with 1 degree of freedom (see Results). In all further analyses, we pooled males' and females' exploratory behavior scores and fitted sex as a fixed effect.

We first estimated the heritability of exploratory behavior for the entire dataset. We then estimated the heritability in juveniles (<12 months) and in adults (>12 months) separately, by running two univariate animal models with the same structure as previously described, and with brood identity fitted as an additional random effect for juveniles. To calculate the genetic correlation between juveniles (<12 months, measured before the breeding season) and adults, we fitted a bivariate mixed model with exploratory behavior in juveniles and in adults fitted as two response variables and similar fixed and random effects. Brood identity was fitted as a random effect for juvenile exploratory behavior only, and covariances on the year, observer, and residual levels were assumed to be zero (diagonal matrices). Hence, only the genetic covariance between juveniles and adults was estimated. We tested whether this covariance was statistically different from 0 (or the correlation different from 1) by comparing this model to models where the additive genetic covariance across ages was assumed to be zero (or the correlation was fixed to 1) using LRT with one degree of freedom. Finally, we tested whether additive genetic variances were different between the two age classes by comparing this model to a model where both variances were constrained to be the same using LRT with one degree of freedom.

### 2.5 Random‐regression animal model

A random‐regression animal model (RRAM; Wilson, Charmantier, & Hadfield, 2008) is an approach to capture a **G** matrix with many character states into a simplified format requiring fewer genetic parameters to be estimated. We used RRAM to test whether additive genetic variance changes with age. The RRAM was a univariate mixed model in which the exploratory behavior score of the individual *i* was modeled as a function of age (*age*) as follows:

$${LOCOM}_{i,age}=\mu +{AGE}_{F}+Sex+Date+{Date}^{2}+f_{A}\left(x,age\right)+{Year}_{age}+Observer+e_{i,age}$$

where *μ* is the overall fixed effect mean, AGE_F_ is a factorial fixed effect capturing the variation in average exploratory behavior across all age groups. The random regression function ***f*_A_(*x,* age)** is an orthogonal polynomial of order *x* on the additive genetic level and captures deviation from the mean effect of Age (Henderson, 1982). The presence of G×A can be tested by comparing a model where *x* = 0 (only variance in intercepts is estimated) to a model where *x* = 1 (variance in intercepts, slopes and their covariance are estimated) using LRT with 2 degrees of freedom. If the latter is significantly better than the former, one can then proceed to increase the order of the polynomial *x* and perform LRT with the appropriate number of degrees of freedom at each step until there is no more significant improvement in the model fit (cf. Brommer, Rattiste, & Wilson, 2010).

For these analyses, we defined different age classes based on individual's age in years and pooled individuals of 3 years old and older. This population therefore consists of 4 age classes: “0–1” (<12 months), “1–2” (12–24 months), “2–3” (24–36 months), and “3+” (>36 months). Age was grand mean centered in these analyses. Residual variance is likely to vary across age groups, and therefore, residuals were fitted as heterogeneous across age classes. Heterogeneity in residuals across age classes was tested by comparing this model to a model where only one residual variance was estimated using LRT with 3 degrees of freedom. The between‐year variance was also allowed to vary across age classes, and heterogeneity in year variance was tested using a similar approach. In contrast to the other variance components, observer variance was fitted as homogeneous. Along with sex and age, date of measurement was fitted as a quadratic fixed effect.

Based on the most parsimonious RRAM, we computed age‐specific heritability (additive genetic variance divided by the sum of additive genetic and residual variances) and genetic correlations between ages. The standard errors of age‐specific heritability and genetic correlations were approximated using the delta method (Fischer, Gilmour, & Werf, 2004). All statistical analyses were performed in R (R Development Core Team, 2018).

## 3 RESULTS

### 3.1 Absence of genotype–sex interactions

Bivariate animal models showed that the genetic correlation between sexes (*r*_g_ = 0.78, *SE* = 0.35) was statistically different from zero (*χ* ^2^ = 4.49, *df* = 1, *p* = 0.034) and not different from 1 (*χ* ^2^ = 0.24, *df* = 1, *p* = 0.62). In addition, sex‐specific variances on all levels did not differ significantly between the sexes (Table S3). We hence jointly considered males and females in further analyses.

### 3.2 Summary statistics

Sample size, average, and standard deviation of exploratory behavior for each age group are reported in Table 1. In this population, the average exploratory score increased from 0 to 2 years old before stabilizing at a slightly lower score in old (>3 years) age classes.

**Table 1:** Sample size, mean and standard deviation (*SD*) for exploratory behavior at each age and for pooled ages (“3,4,5 combined,” “all adults”)

Age	*n*	Mean	*SD*
0	3,984	9.52	7.56
1	645	11.89	8.75
2	167	16.08	9.48
3	73	12.26	8.09
4	33	15.55	8.57
5	9	15.44	9.44
3,4,5 combined	115	13.45	8.41
All adults	927	12.84	8.98

### 3.3 Population‐level plasticity and heritability in exploration

The univariate animal model with all ages and sexes pooled showed that exploratory behavior was heritable in this population (*h^2^* = 0.17, *SE* = 0.04) and that there was a significant quadratic effect of age and season but no significant difference between the sexes (Table 2). There was also a significant year and observer variance. Univariate models in juveniles and adults separately showed that exploratory behavior was significantly heritable in juveniles (*h^2^* = 0.31, *SE* = 0.06, CV_A_ = 6.96, LRT test: *χ* ^2^ = 21.95, *df* = 1, *p* < 0.001) but not significantly heritable in adults (*h* ^2^ = 0.15, *SE* = 0.11, CV_A_ = 3.32, LRT test: *χ* ^2^ = 2.59, *df* = 1, *p* = 0.11) (Tables S1 and S2). Heritability and CV_A_ in adults were both approximately half of heritability and CV_A_ in juveniles, which suggests a reduction in additive genetic variance in adults.

**Table 2:** Fixed and random effects (and their standard error [*SE*]) estimated by the univariate mixed model for exploratory behavior. The significance of fixed effects was tested using conditional Wald *F* tests. Coefficients for males and females are expressed as contrasts and specify the difference between males' and females' average score relative to individuals with unknown sex

Effect	Estimate	*SE*	Test	*p* Value
Random effects				
Year	11.59	4.06	*χ* ^2^ = 144.05	<0.001
Observer	1.89	0.74	*χ* ^2^ = 34.02	<0.001
Additive genetic	8.18	2.01	*χ* ^2^ = 17.57	<0.001
Residual	41.29	2.03		
Fixed effects				
Intercept	6.74E+00		*F*_1,31.2_ = 56.67	<0.001
June days	6.44E−02		*F*_1,3878.5_ = 139.30	<0.001
June days^2^	−1.59E−04		*F*_1,3794.3_ = 106.40	<0.001
Age	1.87E−01		*F*_1,5030.0_ = 28.96	<0.001
Age^2^	−2.88E−03		*F*_1,5060.5_ = 17.70	<0.001
Sex			*F*_2,5071.0_ = 1.81	0.16
Female	−7.08E−01			
Male	−4.96E−01			

### 3.4 Genetic correlation between juveniles and adults

The genetic correlation between juveniles' and adults' exploratory behavior derived from the bivariate animal model (Table 3) was moderately positive (*r*_g_ = 0.22, *SE* = 0.36) but not different from 0 (*χ* ^2^ = 0.37, *df* = 1, *p* = 0.54) or 1 (*χ* ^2^ = 1.88, *df* = 1, *p* = 0.17). *V*_A_ was not statistically different between juveniles and adults (*χ* ^2^ = 0.29, *df* = 1, *p* = 0.59) but *V*_R_ was statistically higher in adults than in juveniles (*χ* ^2^ = 9.40, *df* = 1, *p* = 0.003). The reduction in heritability of exploratory behavior in adults compared to juveniles was hence at least partly driven by an increase in residual variance.

**Table 3:** Variance components (and their standard error [*SE*]) estimated by a bivariate animal model with exploratory behavior in juveniles and in adults as two responses variables

Variance component	Estimate	*SE*
Year		
Juveniles	11.47	4.14
Adults	11.00	4.54
Observer		
Juveniles	0.79	0.47
Adults	1.22	1.11
Brood (Juveniles)	0.00	n.e.
Additive genetic		
Juveniles	14.71	2.81
Covariance Juveniles‐Adults	2.77	4.35
Adults	10.29	7.41
Residual		
Juveniles	32.08	2.65
Adults	57.49	7.65

### 3.5 Age‐related changes in variance components based on random regression

**Figure 1:**
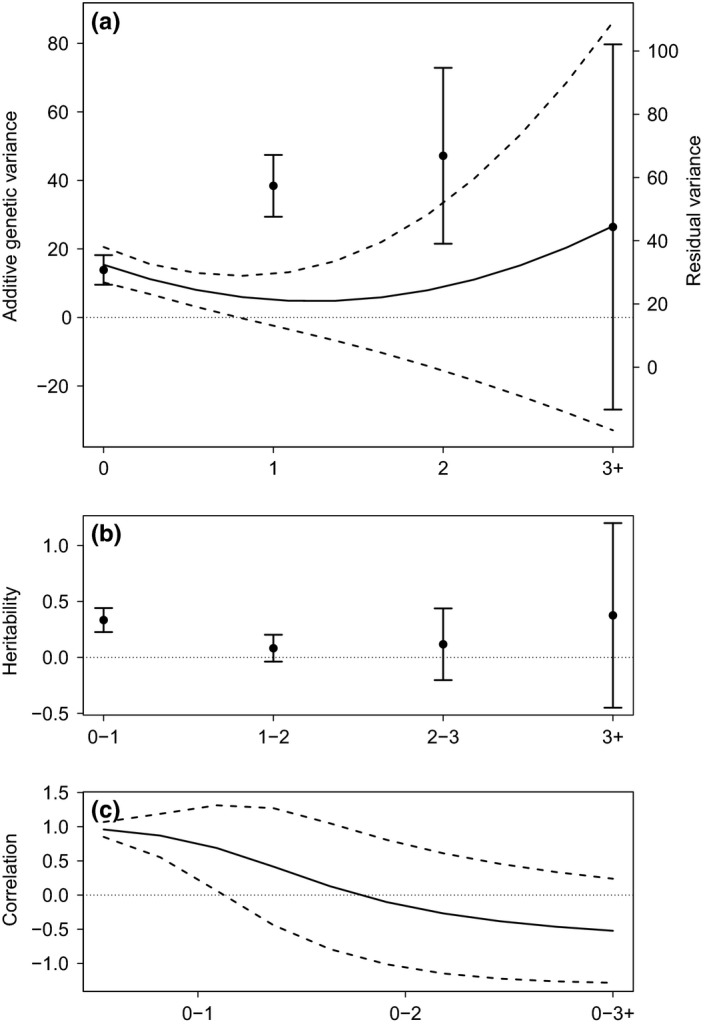
Upper panel (a), left axis: Additive genetic variance (solid line) and its 95% confidence interval (dashed lines) across ages. Upper panel (a), right axis: Dots and whiskers show the age‐specific point estimates and 95% confidence intervals of residual variance. Middle panel (b): Dots and whiskers show point estimates of heritability calculated as the ratio *V*_A_/(*V*_A_ + *V*_R)_ for each age class and their 95% confidence intervals. Lower panel (c): Additive genetic correlation between the first age class (0) and other age classes and its 95% confidence interval (dashed lines)

Comparing models with heterogeneous and homogeneous residuals and year effects indicated heterogeneity in residuals (*χ* ^2^ = 64.28, *df* = 3, *p* < 0.001) and year effects (*χ* ^2^ = 34.16, *df* = 3, *p* < 0.001) across age groups. Comparing the model without G×A (RRAM with *x* = 0) to the model with G×A (RRAM with *x* = 1) suggested that there was significant G×A (*χ* ^2^ = 7.86, *df* = 2, *p* = 0.019). The model with quadratic random slopes did not constitute a significant improvement of the model fit (*χ* ^2^ = 2.01, *df* = 3, *p* = 0.57). Age‐specific variances and their 95% confidence intervals were estimated based on the additive genetic random regression covariance matrix (Table 4) and plotted in Figure 1a. A decrease in *V*_A_ from 0 to 1 year old was clear, followed by an apparent increase in *V*_A_ after 1 year old, although the uncertainty around later age estimates was large and encompassed zero (Figure 1a). Furthermore, *V*_R_ doubled from 0 to 2 years old before declining in older individuals but remained higher in adults than in juveniles (Figure 1a). Based on age‐specific estimates of *V*_A_ and *V*_R_, heritability *h* ^2^ (and standard errors) calculated for each age group was as follows 0.33 (0.05) in 0–1 year old, 0.08 (0.06) in 1–2 years old, 0.12 (0.16) in 2–3 years old and 0.37 (0.41) in older individuals (Figure 1b). Correlation between the first age class and older age classes (Figure 1c) showed a declining pattern. Genetic correlation between 0 and 1 year old was positive (*r*_g0‐1_ = 0.76, *SE* = 0.26) and then essentially reduced to zero at age of 2 years old (*r*_g0‐2_ = −0.16, *SE* = 0.46) and later (Figure 1c).

**Table 4:** Variance components (and their standard error [*SE*]) estimated by a random regression animal model in which additive genetic variance is a linear function of age, while between‐year and residual variances are allowed to differ across the 4 age categories considered

Variance component	Estimate	*SE*
Year		
Age 0–1	7.85	3.03
Age 1–2	9.50	4.12
Age 2–3	15.14	9.88
Age 3+	9.14	9.29
Observer	2.40	0.90
Additive genetic		
Intercept	5.22	6.85
Covariance Intercept‐Slope	2.81	7.46
Slope	15.79	10.02
Residual		
Age 0–1	30.77	2.39
Age 1–2	57.37	4.99
Age 2–3	66.86	14.19
Age 3+	44.36	29.46

### 3.6 Test for selective disappearance

The selective disappearance of individuals between age classes (due to natural selection or dispersal) can cause age‐related changes in means and declines in additive genetic variance for certain traits, when individuals in older age classes are a (biased) sample of the first age class regarding these traits. However, we found that the probability for a juvenile measured in autumn to be caught at least once as an adult (from March of the following year onwards) was not significantly affected by its exploratory behavior (glm with binomial distribution, coefficient = −0.007, *SE* = 0.008, *p* = 0.36).

### 3.7 Simulations

We performed simulations to test probability of detecting G×A when it is absent, and to estimate the power to detect a significant cross‐age genetic correlation in this dataset. In a similar data structure where no G×A was present, the probability of erroneously detecting G×A is only 5% (Text S1). Although these simulations also show the limited power of our dataset to detect heritability of adult exploration and cross‐age genetic correlations, they also indicate that in the absence of G×A, the cross‐age genetic correlation between juveniles and adults would be much higher than what we obtain (mean = 0.78, 95% CI = [0.25; 0.99]).

## 4 DISCUSSION

In this study, we show that heritability of exploratory behavior, a commonly used animal personality measure in various organisms, decreases rapidly during the first year of life because of G×A in a wild population of great tits. Our results add to the few reports of G×A in wild populations and to the even fewer studies investigating this phenomenon in behaviors. We see two major implications of this finding.

First, personality is typically implicitly or explicitly assumed to be constant over ages. Review of literature estimates, however, shows that rank‐order correlations in phenotypic values of behaviors across ontogenetic stages in a wide range of organisms are only about 0.3 (Brommer & Class, 2015). These low correlations suggest that I×A and G×A are common in behaviors, which was supported by empirical work on wild blue tits' behavioral responses to handling (Class & Brommer, 2015, 2016). In the present study, we demonstrate for the first time that G×A occurs in wild great tits' exploratory behavior. Our findings imply that the genetic architecture underlying exploratory behavior in great tits changes as the individual ages leading to a low genetic correlation between exploratory behavior at different ages and a reduction in heritability with age. Such age‐related changes in genetic architecture are particularly important for studies aspiring to find causal genes for behavior; such studies clearly have to adhere to an age‐specific consideration of personality.

Second, our findings have direct implication for understanding the evolutionary dynamics of personality. This is because when G×A is present, evolutionary changes in personality will strongly depend on when during the life cycle of the organism selection in operating. In particular, the strong age‐related decrease in *V*_A_ of exploratory behavior we here document implies that selection on exploration in adults (when heritability is low and nonsignificant) makes little evolutionary impact, whereas selection on exploration directly after fledging (when heritability is high and significant) is expected to clearly impact evolutionary responses. In passerines, the majority of fledged offspring does not recruit into the breeding population, and there is hence clear scope for selection at the youngest age class. Although we do not find evidence for selective disappearance based on juveniles' exploration (directional selection), our results do not rule out fluctuating (across years) and sex‐specific selection, which was previously shown for adults' exploration in this population (Dingemanse, Both, Drent, & Tinbergen, 2004). In addition, earlier findings of a positive relationship between exploration and natal dispersal in this population (Dingemanse, Both, Noordwijk, Rutten, & Drent, 2003) and in another population (Korsten et al., 2013) suggest that a considerable evolutionary force shapes early‐life exploration behavior in great tits.

Immigrants in this population indeed have a higher exploration score than local recruits (Dingemanse et al., 2003). Because these individuals are mainly birds that disperse before their first breeding attempt and because both immigrants and local recruits are equally represented in all age classes (Table S4), this process cannot have caused the G×A pattern that we observe. In addition, although the immigration of fast exploring adults (>1 year old) could drive an increase in mean exploratory behavior of adults compared to juveniles, it would not explain the observed decrease in *V*_A_ of exploratory behavior in adults. This is because variance would increase under this scenario as nondispersing local adults would carry genes for slow exploration and dispersing immigrants for fast exploration.

Over‐reliance on RRAM to test for G×A has been previously criticized because of the lack of consensus in model fitting procedures and restrictive assumptions which can generate unreliable results (Charmantier et al., 2014). However, the low cross‐age correlation estimated by the bivariate animal model aligns with our findings and simulations confirm that our results are robust and not influenced by the data structure (in which adults are less represented than juveniles).

To conclude, we find age‐related plasticity as well as genetic variation in age‐related plasticity in exploratory behavior of wild great tits thereby providing evidence for G×A in personality. Because G×A is likely to be common, not only in life history but also in behavioral traits, and can have consequences for evolutionary predictions, we hope our study will draw more attention to this phenomenon in the future.

## CONFLICT OF INTEREST

None declared.

## AUTHOR CONTRIBUTIONS

All authors conceived the study. KvO collected and compiled the data. BC performed the analyses and wrote the manuscript with inputs from JEB and KvO. All authors approved the final version of the manuscript.

## ETHICAL STATEMENT

Capturing birds, taking blood samples, and the behavioral testing procedures were approved by the Institutional Animal Care and Use Committee: the Royal Academy of Sciences—Animal Experiment Committee (KNAW‐DEC licenses CTO 00.02, CTO 07.03, NIOO 10.05, and NIOO 14.11 to KVO).

## Data Availability

Data files and R scripts supporting the results are accessible on Dryad (https://doi.org/10.5061/dryad.f5d2070).
