# Supplementary material for: Exploratory behavior undergoes genotype–age interactions in a wild bird
Source: Ecol Evol. 2019 Jul 31;9(16):8987–94. doi: 10.1002/ece3.5430 (PMC6706179; doi:10.1002/ece3.5430)
Supplement: Supplementary file 1 [file ECE3-9-8987-s001.docx]

Supplementary material for “Exploration behavior undergoes genotype-age interactions in a wild bird”

Class B., Brommer JE., van Oers K.

**Table S1:** Fixed and random effects (and their standard error SE) estimated by the animal model for exploratory behavior in juveniles. The statistical significance of random effects tested using LRT with 1df. The significance of fixed effects was tested using conditional Wald-F tests. Coefficients for males and females are expressed as contrasts and specify the difference between males’ and females’ average score relative to individuals with “unknown” sex.

| **Effect** | **Estimate** | **SE** | **Test** | **p.value** |
| --- | --- | --- | --- | --- |
| **Random effects** | |  |  |  |
| Year | 11.52 | 4.155 | χ2=59.06 | < 0.001 |
| Observer | 0.78 | 0.464 | χ2=14.22 | < 0.001 |
| Brood | 0.00 | 0.000 | χ2<0.001 | 1.00 |
| Additive genetic | 14.67 | 2.810 | χ2=21.95 | < 0.001 |
| Residual | 32.13 | 2.649 |  |  |
|  |  |  |  |  |
| **Fixed effects** | |  |  |  |
| Intercept | 5.13E+00 |  | F_1,110.1_ = 16.55 | < 0.001 |
| June.days | 1.25E-02 |  | F_1,3648.8_=0.62 | 0.43 |
| June days^2^ | 2.32E-05 |  | F_1,3732.1_=0.14 | 0.71 |
| Age | 1.45E+00 |  | F_1,3687_=5.60 | 0.02 |
| Age^2^ | -1.00E-01 |  | F_1,3840.9_=3.30 | 0.07 |
| Sex |  |  | F_2,3806.8_=0.69 | 0.69 |
| female | -3.09E-01 |  |  |  |
| male | -1.67E-01 |  |  |  |

**Table S2:** Fixed and random effects (and their standard error SE) estimated by the animal model for exploratory behavior in adults. The significance of fixed effects was tested using conditional Wald-F tests. Coefficients for males and females are expressed as contrasts and specify the difference between males’ and females’ average score relative to individuals with “unknown” sex.

| **Effect** | **Estimate** | **SE** | **Test** | **p.value** |
| --- | --- | --- | --- | --- |
| **Random effects** | |  |  |  |
| Year | 11.08 | 4.565 | χ2=44.53 | < 0.001 |
| Observer | 1.23 | 1.117 | χ2=5.63 | 0.02 |
| Additive genetic | 10.45 | 7.409 | χ2=2.59 | 0.11 |
| Residual | 57.32 | 7.647 |  |  |
|  |  |  |  |  |
| **Fixed effects** | |  |  |  |
| Intercept | 6.28E+00 |  | F_1,853.1_ = 2.67 | 0.1 |
| June.days | 8.50E-02 |  | F_1,604.4_=28.93 | < 0.001 |
| June days^2^ | -2.18E-04 |  | F_1,631.2_=26.36 | < 0.001 |
| Age | 2.59E-01 |  | F_1,908.9_=4.60 | 0.03 |
| Age^2^ | -3.85E-03 |  | F_1,906.3_=5.20 | 0.02 |
| Sex |  |  | F_2,900.3_=0.42 | 0.66 |
| female | -3.07 |  |  |  |
| male | -2.92 |  |  |  |

| **Component** | **Parameter** | **Estimate** | **SE** | **z.ratio** | **df** | $\boldsymbol{\chi}_{\boldsymbol{1}}^{\boldsymbol{2}}$ | $\boldsymbol{pval}_{\boldsymbol{1}}$ | $\boldsymbol{\chi}_{\boldsymbol{2}}^{\boldsymbol{2}}$ | $\boldsymbol{pval}_{\boldsymbol{2}}$ |
| --- | --- | --- | --- | --- | --- | --- | --- | --- | --- |
| **Year** | V_m_ | 8.04 | 3.02 | 2.66 | 1 | 0.39 | 0.53 |  |  |
|  | V_f_ | 11.33 | 4.03 | 2.81 |  |  |  |  |  |
| **Observer** | V_m_ | 1.53 | 0.82 | 1.88 | 1 | 0.61 | 0.43 |  |  |
|  | V_f_ | 0.70 | 0.46 | 1.55 |  |  |  |  |  |
| **Additive genetic** | V_m_ | 11.22 | 3.98 | 2.82 | 1 | 0.6 | 0.43 |  |  |
|  | V_f_ | 6.64 | 3.34 | 1.99 |  |  |  |  |  |
|  | COV_mf_ | 6.70 | 2.67 | 2.51 | 1 | 4.49 | 0.034 | 0.24 | 0.62 |
| **Residual** | V_m_ | 42.07 | 3.98 | 10.58 | 1 | 0.03 | 0.85 |  |  |
|  | V_f_ | 43.19 | 3.44 | 12.54 |  |  |  |  |  |

**Table S3:** Variances and covariances, their standard error (SE), and z ratio, estimated by a bivariate animal model with exploratory behavior in males and in females as two responses variables. For each component, we tested whether male and female variances differ ($\chi_{1}^{2}$and ${pval}_{1}$) or whether there covariance differs from 0 ($\chi_{1}^{2}$and ${pval}_{1}$) or 1 ($\chi_{2}^{2}$and ${pval}_{2}$) using LRT with 1 degree of freedom (df).

**Table S4:** Distribution of local and immigrant birds in the different age classes ringed and tested (0 = ringed as nestling; 1= 0-12 months; 2= 12-24 months; 3=>24 months; UNK=unknown age)

|  | **Locals** | | | **Immigrants** | | | |
| --- | --- | --- | --- | --- | --- | --- | --- |
|  | **Age class test** | | | | | | |
| **Age class ring** | 1 | 2 | 3 | 1 | 2 | 3 | UNK |
| **0** | 1861 | 330 | 93 |  |  |  |  |
| **1** |  |  |  | 2434 | 331 | 162 |  |
| **3** |  |  |  |  |  | 80 |  |
| **UNK** |  |  |  |  |  | 27 | 1058 |

**Text S1:** Simulations

We simulated data for a trait which does not undergo GxA 1000 times using our data structure and the package pedantics. In these simulated data sets, VA and VR of the trait are similar to the variances estimated in the univariate animal model for exploration with all ages pooled. We then run RRAM, univariate, and bivariate animal models on this data:

Firstly, we counted how often we find GxA using RRAM to see if a lack of data in older ages can generate type I error. The probability of finding GxA using RRAM is only 5% (n=495). This means that that finding GxA when it does not occur is a rare event, and our findings are not influenced by older individuals having lower pedigree quality than juveniles.

Secondly, we tested the power to detect heritability in adult exploration. In situations without GxA, heritability in adults is slightly higher than what we find (mean=0.21, 95%CI= [0.07; 0.39], n=761) but the probability to statistically detect it is only 32% which implies low power to detect heritability in adult exploration.

Finally, we tested the power of the character-state approach to detect a positive cross-age genetic correlation. We find that in situations without GxA where the correlation across ages is 1, there is a 62% chance of statistically detecting a cross-age genetic correlation different from zero. This again means that power of the character-state approach using this dataset is limited to detect genetic correlations across age classes. However, on average, estimates of this correlation are much higher than what we obtained in our study (mean=0.78, 95%CI=[0.25 ; 0.99],n=761).
